# Biomechanical Analysis of Truncated Cone Implants for Maxillary Sinus Lift: An In Vitro Study on Polyurethane Laminas

**DOI:** 10.3390/bioengineering12010053

**Published:** 2025-01-09

**Authors:** Luca Comuzzi, Tea Romasco, Carlos Fernando Mourão, Giulia Marchioli, Adriano Piattelli, Natalia Di Pietro

**Affiliations:** 1 Independent Researcher, 31020 San Vendemiano, Italy; luca.comuzzi@gmail.com; 2 Department of Medical, Oral and Biotechnological Sciences, “G. D’Annunzio” University of Chieti-Pescara, 66100 Chieti, Italy; giuliamarchioli912@gmail.com (G.M.); natalia.dipietro@unich.it (N.D.P.); 3 Center for Advanced Studies and Technology (CAST), “G. D’Annunzio” University of Chieti-Pescara, 66100 Chieti, Italy; 4 Division of Dental Research Administration, Tufts University School of Dental Medicine, Boston, MA 02111, USA; 5 Department of Clinical and Translational Research, Tufts University School of Dental Medicine, Boston, MA 02111, USA; carlos.mourao@tufts.edu; 6 School of Dentistry, Saint Camillus International University of Health and Medical Sciences, 00131 Rome, Italy; apiattelli51@gmail.com; 7 Facultad de Medicina, UCAM Universidad Católica San Antonio de Murcia, 30107 Murcia, Spain

**Keywords:** dental implants, maxillary sinus lift, truncated cone implants, morse cone connection, polyurethane lamina, implant primary stability

## Abstract

This study aimed to evaluate the biomechanical performance of two truncated cone implant designs in maxillary sinus lift (MSL) procedures using polyurethane laminas. A total of 128 implants were used. Polyurethane laminas were divided into two groups based on thickness (1 and 3 mm) and two subgroups based on density (20 and 30 pounds per cubic foot, PCF). Each subgroup tested two implants (Sinus-plant and Sinus Lift Concept: SLC), resulting in 8 experimental conditions and 16 implants per condition. The insertion torque (IT), removal torque (RT), and implant stability quotient (ISQ) were measured. SLC implants achieved significantly higher IT and RT across all tested conditions (*p* < 0.0001), reporting the highest values at the 30 PCF/3 mm lamina (IT: 34.09 ± 0.32 Ncm; RT: 32.15 ± 0.29 Ncm) and the lowest at the 20 PCF/1 mm lamina (IT: 11.86 ± 0.22 Ncm; RT: 10.28 ± 0.22 Ncm). Additionally, SLC implants achieved significantly higher ISQ values, ranging from around 61 to 48 ISQ. Notably, this difference was not significant at the 20 PCF/3 mm lamina, highlighting that bone density may play a more critical role than thickness for SLC implants. This study simulated the clinical condition of achieving primary stability even with extreme maxillary bone thickness. The findings indicate that while both implant designs can be utilized in MSL procedures, the SLC is particularly effective in scenarios with limited bone thickness and density, potentially allowing for simultaneous MSL, implant placement, and healing screw application.

## 1. Introduction

Maxillary sinus lift procedures are essential to dental implantology, particularly in cases with insufficient residual bone height (RBH) in the posterior maxilla. Ensuring the stability and success of implants in this region is paramount, with implant design, surgical technique, and bone quality playing a crucial role in achieving these objectives [1,2]. Specifically, several surgical approaches have been proposed to increase RBH in the posterior maxilla for the insertion and integration of dental implants. The choice between a crestal or vertical approach depends on the vertical RBH, marginal bone width, local intrasinus anatomy, and the number of teeth to be replaced. The transcrestal approach is suitable for single tooth gaps with adequate width for implant placement and a minimal RBH of 5 mm or for minor augmentation. This procedure generally involves inserting grafting materials immediately before implant placement. In contrast, a lateral sinus floor elevation is recommended when the RBH is less than 5 mm. In particular, simultaneous implant placement is advised when the RBH exceeds 3 mm or several teeth need to be replaced. Instead, a two-stage lateral sinus lift (LSL) with grafting and implant placement should be performed separately when the RBH is less than 3 mm [1,3]. 

Over time, lateral techniques first described by Tatum, Boyne and James, and Kent and Block [4,5,6] using autologous bone grafts for bone augmentation have been replaced or combined with alternative techniques using biological or synthetic grafts, with different healing times based on the graft material used [7,8,9]. Moreover, various minimally invasive strategies have also been developed to optimize LSL results, enhance patient satisfaction, shorten healing times, and reduce costs. These include antral membrane balloon elevation, short-length implants, and the use of osseodensification drills to perform a one-stage LSL with implant placement [10,11,12]. Although studies on LSL and simultaneous implant placement remain sparse, the findings have shown no significant differences in implant survival rate and graft dimensional changes for different grafting materials or compared to the two-stage technique [13,14,15]. However, immediate implant placement is often associated with a risk of early implant failure or marginal bone resorption over time if inadequate primary implant stability is achieved [16]. Therefore, modifying the implant design, connection, surgical technique, and bone quality can improve primary stability [17,18,19]. 

Recently, a truncated cone implant macro-morphology has been tested in vitro and in vivo for extreme sinus rehabilitation as it generates progressive expansion in the bone without stressing it [20,21]. Specifically, a self-condensing truncated cone implant with a specific thread design (Sinus Lift Concept: SLC) has been developed to enhance primary stability in 1–3 mm RBH within the posterior maxilla. This aims to favor a simultaneous implant placement with lateral sinus augmentation, streamlining surgery time, reducing costs, and enhancing the stabilizing effect on the graft and blood clot during sinus rehabilitation [22]. However, the biomechanical performance of this design in sinus lift procedures requires thorough evaluation. 

For this reason, this in vitro investigation focused on assessing biomechanical parameters such as the insertion torque (IT), removal torque (RT), and implant stability quotient (ISQ) of this innovative SLC design compared to a different truncated cone implant used as the control (Sinus-plant; Oralplant Suisse, Mendrisio, Switzerland) on polyurethane laminas mimicking extreme maxillary sinus conditions. The primary objective was to identify the most effective truncated cone implant design for maxillary lateral sinus floor elevation procedures and provide insights into the interaction between implant macro-morphology and different sinus thicknesses and densities. Understanding these interactions and simulating the clinical condition of achieving primary stability even with very thin bone thicknesses is vital for optimizing implant selection and allowing for implant placement, LSL, and healing screw application in a single stage, improving clinical outcomes in patients requiring maxillary sinus augmentation. This research not only contributes to the existing body of knowledge but also offers practical guidelines for clinicians in selecting the appropriate implant design based on the specific characteristics of the maxillary sinus environment.

## 2. Materials and Methods

### 2.1. Polyurethane Foam Sheets

The use of solid rigid polyurethane foam blocks and laminas serves as reliable in vitro models for simulating bones with varying densities and properties, thereby facilitating controlled and reproducible testing environments [23]. Sheets with a thickness of less than 3 mm are particularly valuable for simulating recurring critical clinical conditions, such as ridge atrophy and maxillary sinus pneumatization [20]. Notably, this study addressed the challenge of stabilizing an implant in scenarios of reduced bone height (1–3 mm), a situation that is not always manageable with commercially available implants. In instances where the maxillary bone thickness is as minimal as 1–2 mm, the bone typically exhibits a consistency of at least 30 pounds per cubic foot (PCF) due to the predominance of cortical bone. Conversely, at a thickness of 3 mm, there may be 1 mm of low-density bone (20 PCF) interposed between the two cortical layers. This investigation aimed to simulate these conditions as accurately as possible using polyurethane, selecting consistencies of 20 and 30 PCF that closely mimic the ideal clinical circumstances. While these simulations do not replicate real-life conditions entirely, they provide valuable insights into implant stabilization under such challenging scenarios.

Thus, for this assessment, 1- and 3-mm thick laminas with densities of 20 and 30 PCF and dimensions of 130 mm × 180 mm (Sawbones Europe AB, Malmö, Sweden) were utilized to replicate the thickness and quality of maxillary bone in extreme atrophic conditions. The 20 and 30 PCF density laminas correspond to densities of 0.32 g/cm^3^, similar to the D2 bone type, and 0.48 g/cm^3^, resembling the D1 bone type, respectively, based on the Misch classification [24]. 

### 2.2. Implant Characteristics

The two implants examined in this study were previously described and compared using various rigid polyurethane foam blocks to simulate maxillary sinus rehabilitation [22]. Briefly, both implants are designed with truncated cone macro-morphologies aimed at minimizing stress, promoting progressive bone expansion, and facilitating osseodensification, thereby enhancing the stabilization of the implant at the coronal portion under conditions of reduced bone height. This design may facilitate simultaneous implant placement with sinus lift procedures, effectively reducing the number of surgical interventions from three to one. Additionally, they incorporate a titanium-lapped round apex to preserve the Schneiderian membrane and create controlled insertion pressure, along with a conometric coupling with a switching platform that helps limit marginal bone level alteration and offers improved sealing to reduce bacterial colonization compared to other connections [25,26]. 

**Figure 1:**
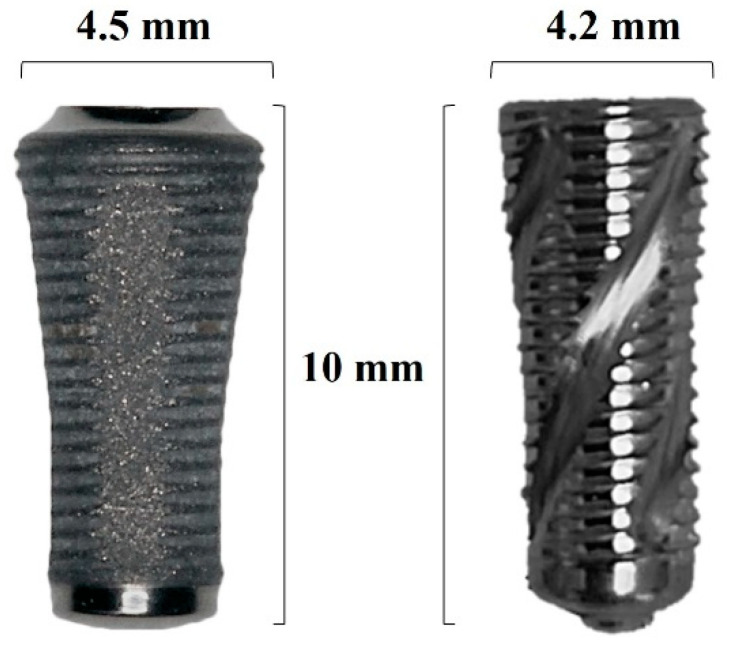
Implant designs utilized in the current study. Sinus-plant implant (Oralplant Suisse, Mendrisio, Switzerland) on the left side, and Sinus Lift Concept implant (SLC, AoN Implants Srl, Grisignano di Zocco, Italy) on the right side.

The SLC implants (AoN Implants Srl, Grisignano di Zocco, Italy) have a diameter of 4.2 mm and a length of 10 mm, a machined surface, a self-tapping V-shaped 1.17 mm pitch profile with an 11° thread angle, and a progressive and expansive helicoidal grooves from the apex to the platform. These latter facilitate debris accumulation at the area and defuse blood during implant insertion, fostering an osteopromotive environment and proper implant osseointegration, while reducing the risk of interference with primary stability [27,28]. Conversely, the Sinus-Plant implants (Oralplant Suisse, Mendrisio, Switzerland) presented a diameter of 4.5 mm and a length of 10 mm, with a 0.4 mm pitch profile of the threads, longitudinal grooves, and a titanium pull spray superficial (TPSS) treatment that provides rounded and porous microcavities on the implant surface. Despite the slight differences in diameter, we chose the most closely matched dimensions for comparison due to the manufacturers not producing implants with identical diameters. Moreover, the reduced coronal diameter of the SLC implant mitigates stress on the bone during dilation when the implant is placed, which is particularly advantageous in cases of initially narrow bone (Figure 1).

### 2.3. Implant Drill and Experimental Design

The experimental protocol involved the utilization of a standardized implant drilling procedure conducted by a single operator (LC) for all implant types. This included the use of a 2 mm lance drill, followed by 2.2 mm and 3.2 mm drills (AoN Implants Srl, Grisignano di Zocco, Italy), driven by a surgical implant motor (Chiropro, Bien Air, Bienne, Switzerland) set at 100 rpm.

**Figure 2:**
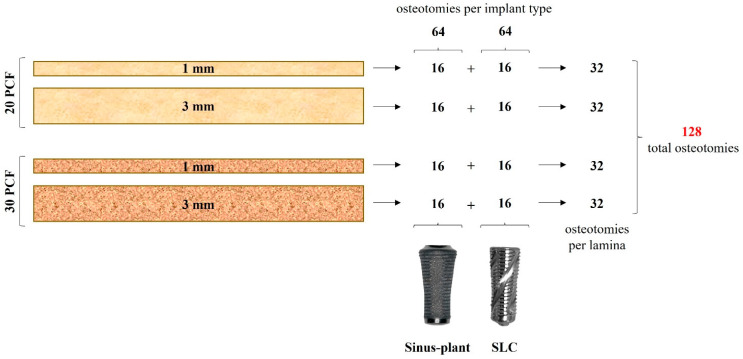
Summary of the experimental design. PCF: pounds per cubic foot.

A total of 128 osteotomies were performed, with 16 conducted for each implant type on polyurethane foam laminas, resulting in 64 osteotomies for each type of implant. In this way, 32 drilling sites were established for each sheet, as depicted in Figure 2.

### 2.4. Insertion Torque (IT) and Removal Torque (RT)

In the assessment of implant stability and osseointegration, the application of straightforward and clinically relevant non-invasive tests is highly beneficial. Among the non-destructive protocols outlined in the literature, elevated IT plays a pivotal role in establishing primary stability by minimizing implant micromotion, which is crucial for predictable implant osseointegration. Implants are typically inserted with forces ≥ 30 Ncm into both healed ridges and fresh extraction sockets to enable immediate loading. Moreover, higher IT (≥50 Ncm) serves to minimize micromotion without causing bone damage [29]. The RT test evaluates the healing capacity at the bone–implant interface by assessing interfacial shear strength through the application of load parallel to the bone–implant interface. For an implant to be considered stable, the RT should exceed 20 Ncm [30].

The implants were positioned at the crest level using a speed of 30 rpm and a predetermined maximum torque of 40 Ncm. Subsequently, using a calibrated torque meter, the IT and RT values were measured at the final millimeter of the implant site.

### 2.5. Resonance Frequency Analysis (RFA)

Resonance frequency analysis (RFA) is a non-invasive diagnostic technique employed to assess implant stability and bone density by utilizing vibration and structural analysis principles. In our case, it involved the use of a specialized device (Smartpeg n.78) and a frequency response analyzer (Osstell Beacon, Ostell Inc., Göteborg, Schweden) to measure the ISQ in two different orientations at 90 degrees: mesiodistal (MD) and buccolingual (BL). The RFA captured ranges from 3000 to 8500 Hz, translating to an ISQ value between 0 and 100. This allows the categorization of implant primary stability as low (less than 60 ISQ), medium (60–70 ISQ), and high (greater than 70 ISQ) based on the obtained ISQ. The device features a graphical display panel presenting MD and BL-ISQ values, indicating the firmness at the bone–implant interface. Higher RFA values indicate a stronger bone–implant interface. Used during implant placement, RFA provides a baseline reading for future comparisons and postsurgical evaluations of the implant. RFA has widespread application in clinically assessing osseointegration and for prognostic evaluation purposes [30].

**Figure 3:**
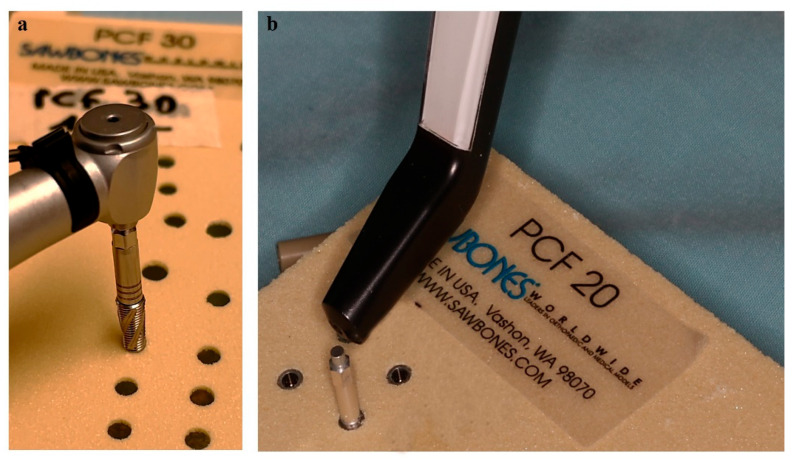
Representative images of the insertion and evaluation of the implant stability. (**a**) Insertion of an SLC implant into a 1-mm thick lamina with a density of 30 PCF using a surgical motor (Chiropro, Bien Air, Bienne, Switzerland). (**b**) Evaluation of the implant stability quotient (ISQ) in the mesiodistal (MD) orientation of an SLC implant subsequent to its insertion into a 1-mm thick lamina with a density of 20 PCF.

In Figure 3, the processes of implant insertion (Figure 3a) and measurement of the ISQ (Figure 3b) are depicted.

### 2.6. Statistical Analysis

The sample size calculation and power analysis were performed utilizing the G*Power 3.1.9.4 program (Heinrich Heine Universität Düsseldorf, Düsseldorf, Germany). The analysis involved the F tests family and a “fixed effects, special, main effects, and interactions analysis of variance (ANOVA)” with an effect size of 0.25, α err of 0.05, power (1-β) of 0.8, numerator df of 1, and 8 groups. The dependent variables for the sample size calculation included IT, RT, BL-RFA, and MD-RFA measurements. In contrast, the independent variables were the implant type (Sinus-plant and SLC), bone density (20 and 30 PCF), and polyurethane lamina thickness (1 and 3 mm). The statistical significance was set at *p*-values ≤ 0.05, with a minimum sample size requirement of 128 implants, encompassing 16 samples per group.

Additionally, after verifying the normality of data distribution through the Shapiro–Wilk test, the study utilized a 2 × 2 × 2 factorial design and applied a three-way ANOVA model followed by Tukey’s post-hoc test. Descriptive statistics were proposed as means ± standard deviations (SD). The GraphPad 9.0 software package (Prism, San Diego, CA, USA) was used for statistical analyses.

## 3. Results

### 3.1. IT Analysis

**Figure 4:**
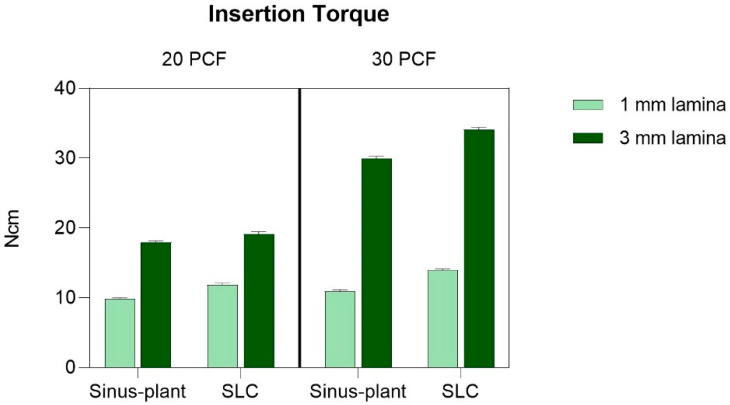
Mean values and standard deviations (SD) of insertion torque (IT) for the two implants tested across the different artificial bone conditions. All multiple comparisons showed statistically significant differences with a *p* < 0.0001.

Table S1 (Supplementary Material S1) provides an overview of the IT results, presenting the confidence interval (CI) and *p*-values for all multiple comparisons among the various experimental conditions. Additionally, Figure 4 illustrates the graphical representations of these comparisons.

The study revealed a strong correlation between the thickness and density of laminas and their IT characteristics. Lower density and lesser thickness in foam laminas resulted in lower IT values, showcasing a more pronounced effect in thicker laminas. Particularly, the lowest IT was observed in the 20 PCF/1 mm lamina for both Sinus-plant and SLC implants (9.82 ± 0.16 Ncm and 11.86 ± 0.22 Ncm, respectively), with Sinus-plant exhibiting significantly lower results. Conversely, the highest IT values were recorded in the 30 PCF/3 mm lamina (29.95 ± 0.33 Ncm and 34.09 ± 0.32 Ncm for Sinus-plant and SLC implants, respectively), with SLC implants demonstrating the highest result. Generally, SLC implants consistently displayed significantly higher values compared to Sinus-plant implants. 

Furthermore, the IT discrepancy between Sinus-plant and SLC implants was lower in the 1 mm thickness laminas with densities of 20 and 30 PCF (9.82 ± 0.16 Ncm vs. 10.93 ± 0.23 Ncm for Sinus-plant implants and 11.86 ± 0.22 Ncm vs. 13.99 ± 0.17 Ncm for SLC implants, respectively) than in thicker laminas (17.91 ± 0.23 Ncm vs. 29.95 ± 0.33 Ncm for Sinus-plant implants and 19.15 ± 0.34 Ncm vs. 34.09 ± 0.32 Ncm for SLC implants, respectively), consistently demonstrating statistical significance. 

Notably, all IT multiple comparisons resulted in statistical significance with a *p* < 0.0001.

### 3.2. RT Analysis

**Figure 5:**
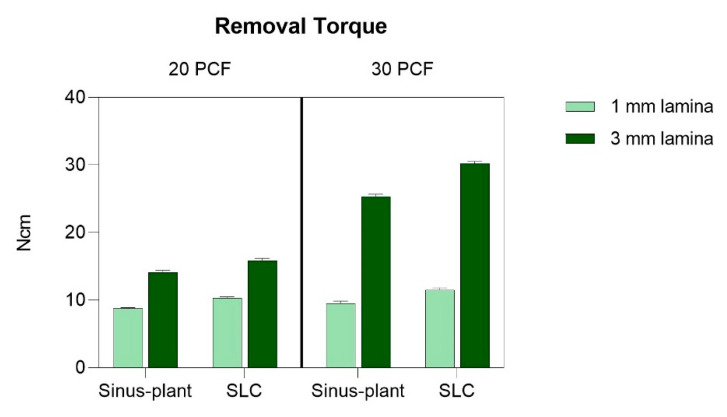
Mean values and SD of removal torque (RT) for the two implants tested across the different artificial bone conditions. All multiple comparisons showed statistically significant differences with a *p* < 0.0001.

Table S2 (Supplementary Material S2) offers a comprehensive summary of the RT results, including CIs and *p*-values for all multiple comparisons across the experimental conditions. Furthermore, Figure 5 provides visual representations of these comparisons.

The RT values exhibited a similar trend with the graphical representation of IT, consistently indicating significantly higher outcomes for SLC implants. The lowest RT values were observed in the 20 PCF/1 mm lamina, measuring 8.77 ± 0.12 Ncm for Sinus-plant implants and 10.28 ± 0.22 Ncm for SLC implants. The highest results were recorded for both implants in the 30 PCF/3 mm lamina, registering at 25.36 ± 0.33 Ncm and 30.23 ± 0.28 Ncm, respectively.

Within the highest-density lamina, differences in RT values expressed by each implant at the two different thicknesses were significantly more pronounced compared to those in the 20 PCF-density lamina. Discrepancies of approximately 16 Ncm and 19 Ncm were observed for Sinus-plant and SLC implants in the highest-density layer, while corresponding values for the 20 PCF density layer were approximately 5 Ncm and 6 Ncm for Sinus-plant and SLC implants, respectively. Furthermore, despite RT being lower than the corresponding IT across all experimental conditions, disparities were only 1–2 Ncm in the 20 PCF/1 mm layer and 3–4 Ncm in the 20 PCF/3 mm layer for both implants. On the other hand, in the highest-density laminas, disparities of 2–3 Ncm in the 1 mm-thick lamina and 4–5 Ncm in the 3 mm-thick lamina were identified.

Moreover, similar to the IT scenario, all multiple comparisons revealed a *p*-value of less than 0.0001.

### 3.3. RFA

Like the IT and RT outcomes, the summary of the CIs and significances pertaining to BL- and MD-RFA measurements has been presented in Tables S3 and S4 (Supplementary Materials S3 and S4).

**Figure 6:**
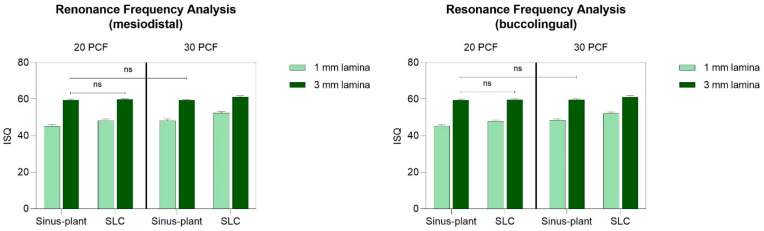
Mean values and SD of ISQ in the MD and buccolingual (BL) orientations for the two implants tested across the different artificial bone conditions. SLC 20 PCF/3 mm vs. SLC 30 PCF/3 mm in both orientations: *p* < 0.001; Sinus-plant 30 PCF/3 mm vs. SLC 30 PCF/3 mm: *p* < 0.001 in the BL orientation; all other multiple comparisons: *p* < 0.0001. ns: not significant.

However, from Figure 6, it can be noted how SLC implants consistently exhibited significantly higher ISQ values compared to Sinus-plant implants in both MD and BL orientations, with a *p* < 0.0001 in most of the cases. This difference was found to be not significant only at the 20 PCF/3 mm lamina and exhibited a *p* < 0.001 at the 30 PCF/3 mm lamina in the BL direction.

In evaluating the stability values, it was observed that discrepancies based on bone density and thickness were more leveled compared to the IT and RT trends. Notably, there were no significant differences between Sinus-plant implants inserted in the 20 and 30 PCF density lamina with a thickness of 3 mm (59.50 ± 0.53 ISQ vs. 59.41 ± 0.52 ISQ in the MD direction and 59.51 ± 0.53 ISQ vs. 59.61 ± 0.52 ISQ in the BL direction), while a statistically significant difference with a *p* < 0.001 was showed between SLC implants inserted in 20 and 30 PCF density lamina with a thickness of 3 mm in both directions (59.80 ± 0.42 ISQ vs. 61.20 ± 0.79 ISQ in the MD direction, and 59.60 ± 0.52 ISQ vs. 61.01 ± 0.94 ISQ in the BL direction). All other comparisons resulted in a significant difference with a *p* < 0.0001.

The highest ISQ values were reported for SLC implants at the 30 PCF/3 mm lamina in both directions (61.20 ± 0.79 ISQ and 61.01 ± 0.94 ISQ in the MD and BL directions, respectively) and for Sinus-plant implant only in the BL direction (59.61 ± 0.52 ISQ). Indeed, the highest values reported by Sinus-plant implants in the MD orientation were at the 20 PCF/3 mm lamina (59.50 ± 0.53 ISQ), which did not differ statistically from the values reported in the 30 PCF/3 mm lamina (59.41 ± 0.52 ISQ). Conversely, the lowest stability results were registered for both implants and in both directions at the 20 PCF/1 mm lamina (45.21 ± 0.79 ISQ and 48.30 ± 0.82 ISQ for Sinus-plant and SLC implants, respectively, in the MD orientation and 45.30 ± 0.82 ISQ and 47.91 ± 0.57 ISQ for the same implants in the BL direction). 

## 4. Discussion

The objective of the current research was to assess the biomechanical performance of two distinct truncated cone implant macro-morphologies—SLC and Sinus-plant implants—under controlled in vitro conditions using polyurethane foam laminas that mimic various maxillary bone densities and thicknesses. The results yielded valuable insights into the influence of implant design on primary stability and osseointegration, both of which are crucial aspects for the success of dental implants, especially in demanding anatomical situations such as severe maxillary bone atrophy with an RBH < 3 mm [31].

The simultaneous implant placement with sinus lift is commonly utilized in cases of insufficient bone volume at the posterior maxilla. However, due to poor bone quality and inadequate bone height, achieving ideal primary stability in this area may be challenging [32]. Consequently, modifications to the implant design, connection, surgical technique, and the quality of bone may enhance primary stability [17,18,19]. Specifically, LSL procedures incorporating autologous bone grafts for augmentation have either been supplanted or supplemented with alternative methodologies that utilize biological or synthetic grafts. These alternatives yield varying healing times relative to the graft material employed, yet demonstrate comparable outcomes concerning implant survival and stability, marginal bone loss, and patient preferences [7,8,9]. Moreover, an array of minimally invasive strategies has been developed to optimize the results of LSL, thereby enhancing patient satisfaction, minimizing healing times, and reducing costs. Such strategies include the introduction of innovative implant designs, including short-length implants, and the utilization of osseodensification drills to facilitate one-stage LSL with concurrent implant placement [10,11,12]. Research by Durrani et al. [11] has indicated that short implants exhibit similar clinical and radiographic performance, including ISQ outcomes, when compared to longer implants placed in conjunction with an LSL, up to 12 months post-prosthetic loading. However, it is important to note that immediate implant placement is often associated with an elevated risk of early implant failure or marginal bone resorption over time, should inadequate primary implant stability be attained [16].

This study revealed significant differences in IT values between the two types of implants across various artificial bone conditions, with SLC implants consistently exhibiting significantly higher results compared to Sinus-plant implants (*p* < 0.0001). The highest IT values were recorded in the 30 PCF/3 mm lamina for SLC implants (34.09 ± 0.32 Ncm) compared to 29.95 ± 0.33 Ncm of Sinus-plant implants. Conversely, the lowest IT was observed in the 20 PCF/1 mm lamina for Sinus-plant implants (9.82 ± 0.16 Ncm) compared to 11.86 ± 0.22 Ncm of SLC implants. This suggests that implant design features such as thread profile and surface treatment play a pivotal role in their mechanical performance [18,33]. The self-tapping V-shaped threads and expansive helicoidal grooves of SLC implants have been shown to significantly improve IT, RT, and primary stability outcomes. These features demonstrated the implants’ capability to accumulate material debris and foster osteopromotive conditions during insertion, resembling clinical scenarios where bone chips play a role in promoting osseointegration. Consequently, this facilitated constant and progressive implant advancement, thereby maintaining the osteotomy cleanliness. Research has validated that the presence of healing niches accelerates short-term osseointegration and sustains it in the long term [34,35], while the thread design of Sinus-Plant implants provided a different interaction with the surrounding bone. Despite previous studies noting that narrower threads could achieve higher bone–implant contact but potentially cause more bone damage, the impact of thread designs on primary stability warrants further investigation [36].

Furthermore, the RFA registered the highest values in the 30 PCF/3 mm lamina for both SLC and Sinus-plant implants (approximately 61 ISQ for SLC implants and 59 for Sinus-plant implants in both orientations), with SLC implants exhibiting significantly higher results and achieving medium stability. Even with a reduction in the lamina’s thickness (30 PCF/1 mm), a similar pattern persisted, and the ISQ decreased to around 52 for SLC implants and 48 for Sinus-plant implants (*p* < 0.0001). It is important to note that in the 20 PCF/3 mm lamina, both implants displayed comparable and consistent values (approximately 60 ISQ in both orientations), suggesting that the density of the polyurethane may have a greater influence than the thickness [37]. These findings align with the existing literature that emphasizes bone density as the primary determinant affecting implant stability in maxillary sinus augmentation procedures with simultaneous implant placement, compared to the impact of RBH. Therefore, preoperative evaluation of bone density may help mitigate stability-related complications in single-stage implant treatments for the atrophic posterior maxilla. Furthermore, while marginal bone stability remains essential, variations in ISQ during the initial healing phases are also significant indicators of long-term success. Specifically, elevated ISQ values in denser bone are associated with more favorable outcomes concerning bone remodeling and stability [38]. Nonetheless, Sinus-plant implants did not show significant differences when comparing 3 mm thick laminas of varying densities (59.50 ± 0.53 ISQ vs. 59.41 ± 0.52 ISQ in the MD direction and 59.51 ± 0.53 ISQ vs. 59.61 ± 0.52 ISQ in the BL direction, at the 20 and 30 PCF laminas, respectively). On the other hand, the lowest ISQ values for both implants were recorded in the 20 PCF/1 mm lamina, at approximately 45 ISQ and 48 ISQ for Sinus-plant and SLC implants, respectively, in both orientations (*p* < 0.0001). From a clinical perspective, a minimum IT of 15 Ncm and ISQ of 65 have been recommended for successful implant osseointegration, with higher values correlating to increased success rates [39]. Overall, SLC implants demonstrated consistent and adequate primary stability even in extreme thickness and density conditions, exhibiting even higher IT with the 3 mm thickness laminas. This suggests a potential correlation between design, surgical technique, and bone density in influencing implant stability [33]. Nevertheless, achieving a very high ISQ is not feasible when the contact area between the polyurethane and the implant ranges from merely 1 to 3 mm. This limitation is understandable; the critical factor remains the assurance of primary stability that is both clinically valid and durable throughout the healing period.

In addition, it appears that the 1 mm under-preparation resulting from using the final drill provides the optimal condition for ensuring good stability across varying bone thicknesses and consistencies. This is evident from the SLC implant values falling within the 48 to 61 ISQ range, which were achieved across all extreme experimental conditions. Consequently, the use of a smaller diameter final drill results in a reduced osteotomy or under-preparation of the site, thereby enhancing both the stability and osseointegration of dental implants, especially in cases of critical bone density and availability [40]. The findings of this study concord with a previously published in vitro investigation involving polyurethane blocks that simulated maxillary sinus rehabilitations [22]. The comparative analysis of the implants revealed that SLC implants demonstrated an optimal level of primary stability exclusively within a 20 PCF density block featuring a 30 PCF density cortical layer, resulting in ISQ values of approximately 70 in both orientations. In contrast, both implants exhibited low primary stability within the 10 PCF density block. However, this outcome was considered acceptable when factoring in the insufficient bone density. In conclusion, SLC implants consistently exhibited significantly superior ISQ values compared to Sinus-plant implants across all experimental conditions, as well as significantly higher IT across all polyurethane densities, with the exception of the lowest-density block.

The use of rigid polyurethane foams as a model to emulate bone density and quality has become a well-established method for evaluating biomechanical tests on dental implants and other medical devices [23]. The laminas employed effectively replicated the mechanical properties of human bone, enabling a controlled assessment of implant performance under different conditions. This approach not only enhances result reproducibility but also establishes a framework for future studies to investigate the biomechanical implications of different implant designs in more complex scenarios. However, it is essential to acknowledge that key factors such as bone metabolism, cell turnover, mineralization, maturation, intercellular matrix, vascularity, and others notably contribute to the definition of bone quality, potentially influencing implant outcomes, as artificial bone cannot fully mimic real bone conditions [41]. Translating these findings to clinical practice requires careful consideration of physiological variables not present in synthetic models. For instance, natural bone undergoes dynamic remodeling influenced by cellular activities, hormonal regulation, and patient-specific factors like age, systemic health, and medication use [42]. Conditions such as osteoporosis or diabetes can significantly alter bone density and healing capacity, potentially affecting implant integration. Furthermore, the presence or absence of vascularization can influence implant stability and the overall success of regenerative materials. Additionally, the impact of the bone graft inserted during the procedure could be considered a limitation of this model, potentially influencing the current findings.

Nonetheless, the clinical implications of these findings are significant with regard to implant design selection, which should be customized based on the patient’s specific anatomical and physiological conditions. For instance, in cases of substantial bone atrophy in the posterior maxilla where achieving primary stability is crucial, the SLC implant may offer advantages due to its design features that enhance interaction with the surrounding bone. Specifically, the advanced thread design and the presence of three bone decompression niches led to the accumulation of polyurethane debris, mimicking a condition that could accumulate bone chips conducive to osseointegration, especially in instances of critical bone thickness. The truncated cone shape of these implants facilitated progressive bone expansion without inducing stress or risking bone laminae fracture, even in cases of critical thickness (1 mm), and allowed for precise and gradual implant advancement under minimal bone stress. Additionally, the slight diameter difference (0.3 mm) maintained similar mechanical friction characteristics as the Sinus-plant implant, providing clinical advantages, such as a smaller final hole diameter and increased versatility in narrow bone platforms. The rounded tip shape of the implant can protect the Schneiderian membrane in the maxillary sinus during implantation, allowing for progressive and controllable elevation, which is particularly useful in bone augmentation procedures in edentulous posterior maxillary regions. This study was conducted to analyze a macro-geometric design specifically developed to enhance implant stability in conditions characterized by reduced bone height. The design facilitates simultaneous implant placement alongside sinus lift procedures and aims to minimize the total number of surgical interventions from three to one. Notably, both implants are designed with a truncated cone shape, with tapers from the widest section at the platform to the narrowest point at the apex, thereby improving the stabilization of the implant in the coronal region. However, it is essential to note that the focus of this investigation is limited to these two specific brands, but the potential for further research to compare them to similar designs from other brands, as well as with alternative macro-designs intended for these clinical conditions, warrants attention [22].

The findings indicated that while both implant designs can be utilized in maxillary sinus lift procedures, the SLC design was particularly effective in scenarios with limited bone thickness and density. In clinical practice, it can be hypothesized that, for the SLC, bone quality may be more important than thickness. Even in cases of very thin bone thickness (1-3 mm, when a vestibular approach for sinus lift is necessary), implants can potentially be placed if the bone has good density. 

## 5. Conclusions

In conclusion, this study emphasizes the significant role of implant macro-morphology in influencing the biomechanical performance of maxillary sinus lift procedures. SLC implants exhibited the capability to stabilize in extremely thin polyurethane layers and demonstrated enhanced features in terms of diameter, threads, and bone decompression niches, thereby improving stabilization in challenging bone conditions and potentially accelerating osseointegration compared to Sinus-plant implants. However, future research should prioritize investigating the long-term outcomes of these implants in clinical settings. Additionally, exploring further design modifications to enhance implant stability and integration is warranted. Overall, these findings contribute to the growing body of evidence supporting the optimization of implant designs to enhance patient outcomes in dental implantology. This evidence may serve as a valuable resource for considering a one-stage surgical approach in situations with limited residual native bone as an alternative to a delayed approach in LSL protocols.

## Data Availability

The data that support the findings of this study are available in the Supplementary Material of this article.

## Supplementary Materials

The following supporting information can be downloaded at https://www.mdpi.com/article/10.3390/bioengineering12010053/s1. Table S1: p-values and confidence intervals (CI) following multiple comparisons of the insertion torque (IT) values across the different experimental conditions; Table S2: p-values and CI following multiple comparisons of the removal torque (RT) values across the different experimental conditions; Table S3: p-values and CI following multiple comparisons of the implant stability quotient (ISQ) values across the different experimental conditions in the buccolingual (BL) direction; Table S4: p-values and CI following multiple comparisons of the ISQ values across the different experimental conditions in the mesiodistal (MD) direction.
