# Supplementary material for: Biomechanical Analysis of Truncated Cone Implants for Maxillary Sinus Lift: An In Vitro Study on Polyurethane Laminas
Source: Bioengineering (Basel). 2025 Jan 9;12(1):53. doi: 10.3390/bioengineering12010053 (PMC11761941; doi:10.3390/bioengineering12010053)
Supplement: Supplementary file 1 [file bioengineering-12-00053-s001.zip › bioengineering-3339595-supplementary/Supplementary file S1.pdf]

Table S1. *P*-values and confidence intervals (CI) following multiple comparisons of the insertion torque (IT) values across the different experimental conditions.

Tukey's multiple comparisons test	95.00% CI of difference	Summary	Adjusted *p*-value
Sinus-plant:20 PCF 1 mm vs. Sinus-plant:20 PCF 3 mm	-8.451 to -7.729	****	<0.0001
Sinus-plant:20 PCF 1 mm vs. Sinus-plant:30 PCF 1 mm	-1.471 to -0.7488	****	<0.0001
Sinus-plant:20 PCF 1 mm vs. Sinus-plant:30 PCF 3 mm	-20.49 to -19.77	****	<0.0001
Sinus-plant:20 PCF 1 mm vs. SLC:20 PCF 1 mm	-2.401 to -1.679	****	<0.0001
Sinus-plant:20 PCF 1 mm vs. SLC:20 PCF 3 mm	-9.691 to -8.969	****	<0.0001
Sinus-plant:20 PCF 1 mm vs. SLC:30 PCF 1 mm	-4.531 to -3.809	****	<0.0001
Sinus-plant:20 PCF 1 mm vs. SLC:30 PCF 3 mm	-24.63 to -23.91	****	<0.0001
Sinus-plant:20 PCF 3 mm vs. Sinus-plant:30 PCF 1 mm	6.619 to 7.341	****	<0.0001
Sinus-plant:20 PCF 3 mm vs. Sinus-plant:30 PCF 3 mm	-12.40 to -11.68	****	<0.0001
Sinus-plant:20 PCF 3 mm vs. SLC:20 PCF 1 mm	5.689 to 6.411	****	<0.0001
Sinus-plant:20 PCF 3 mm vs. SLC:20 PCF 3 mm	-1.601 to -0.8788	****	<0.0001
Sinus-plant:20 PCF 3 mm vs. SLC:30 PCF 1 mm	3.559 to 4.281	****	<0.0001
Sinus-plant:20 PCF 3 mm vs. SLC:30 PCF 3 mm	-16.54 to -15.82	****	<0.0001
Sinus-plant:30 PCF 1 mm vs. Sinus-plant:30 PCF 3 mm	-19.38 to -18.66	****	<0.0001
Sinus-plant:30 PCF 1 mm vs. SLC:20 PCF 1 mm	-1.291 to -0.5688	****	<0.0001
Sinus-plant:30 PCF 1 mm vs. SLC:20 PCF 3 mm	-8.581 to -7.859	****	<0.0001
Sinus-plant:30 PCF 1 mm vs. SLC:30 PCF 1 mm	-3.421 to -2.699	****	<0.0001
Sinus-plant:30 PCF 1 mm vs. SLC:30 PCF 3 mm	-23.52 to -22.80	****	<0.0001
Sinus-plant:30 PCF 3 mm vs. SLC:20 PCF 1 mm	17.73 to 18.45	****	<0.0001
Sinus-plant:30 PCF 3 mm vs. SLC:20 PCF 3 mm	10.44 to 11.16	****	<0.0001
Sinus-plant:30 PCF 3 mm vs. SLC:30 PCF 1 mm	15.60 to 16.32	****	<0.0001
Sinus-plant:30 PCF 3 mm vs. SLC:30 PCF 3 mm	-4.501 to -3.779	****	<0.0001
SLC:20 PCF 1 mm vs. SLC:20 PCF 3 mm	-7.651 to -6.929	****	<0.0001
SLC:20 PCF 1 mm vs. SLC:30 PCF 1 mm	-2.491 to -1.769	****	<0.0001
SLC:20 PCF 1 mm vs. SLC:30 PCF 3 mm	-22.59 to -21.87	****	<0.0001
SLC:20 PCF 3 mm vs. SLC:30 PCF 1 mm	4.799 to 5.521	****	<0.0001
SLC:20 PCF 3 mm vs. SLC:30 PCF 3 mm	-15.30 to -14.58	****	<0.0001
SLC:30 PCF 1 mm vs. SLC:30 PCF 3 mm	-20.46 to -19.74	****	<0.0001
