# Supplementary material for: Biomechanical Analysis of Truncated Cone Implants for Maxillary Sinus Lift: An In Vitro Study on Polyurethane Laminas
Source: Bioengineering (Basel). 2025 Jan 9;12(1):53. doi: 10.3390/bioengineering12010053 (PMC11761941; doi:10.3390/bioengineering12010053)
Supplement: Supplementary file 1 [file bioengineering-12-00053-s001.zip › bioengineering-3339595-supplementary/Supplementary file S2.pdf]

Table S2. *P*-values and CI following multiple comparisons of the removal torque (RT) values across the different experimental conditions.

Tukey's multiple comparisons test	95.00% CI of difference	Summary	Adjusted *p*-value
Sinus-plant:20 PCF 1 mm vs. Sinus-plant:20 PCF 3 mm	-5.786 to -4.974	****	<0.0001
Sinus-plant:20 PCF 1 mm vs. Sinus-plant:30 PCF 1 mm	-1.106 to -0.2937	****	<0.0001
Sinus-plant:20 PCF 1 mm vs. Sinus-plant:30 PCF 3 mm	-17.00 to -16.18	****	<0.0001
Sinus-plant:20 PCF 1 mm vs. SLC:20 PCF 1 mm	-1.916 to -1.104	****	<0.0001
Sinus-plant:20 PCF 1 mm vs. SLC:20 PCF 3 mm	-7.526 to -6.714	****	<0.0001
Sinus-plant:20 PCF 1 mm vs. SLC:30 PCF 1 mm	-3.106 to -2.294	****	<0.0001
Sinus-plant:20 PCF 1 mm vs. SLC:30 PCF 3 mm	-21.87 to -21.05	****	<0.0001
Sinus-plant:20 PCF 3 mm vs. Sinus-plant:30 PCF 1 mm	4.274 to 5.086	****	<0.0001
Sinus-plant:20 PCF 3 mm vs. Sinus-plant:30 PCF 3 mm	-11.62 to -10.80	****	<0.0001
Sinus-plant:20 PCF 3 mm vs. SLC:20 PCF 1 mm	3.464 to 4.276	****	<0.0001
Sinus-plant:20 PCF 3 mm vs. SLC:20 PCF 3 mm	-2.146 to -1.334	****	<0.0001
Sinus-plant:20 PCF 3 mm vs. SLC:30 PCF 1 mm	2.274 to 3.086	****	<0.0001
Sinus-plant:20 PCF 3 mm vs. SLC:30 PCF 3 mm	-16.49 to -15.67	****	<0.0001
Sinus-plant:30 PCF 1 mm vs. Sinus-plant:30 PCF 3 mm	-16.30 to -15.48	****	<0.0001
Sinus-plant:30 PCF 1 mm vs. SLC:20 PCF 1 mm	-1.216 to -0.4037	****	<0.0001
Sinus-plant:30 PCF 1 mm vs. SLC:20 PCF 3 mm	-6.826 to -6.014	****	<0.0001
Sinus-plant:30 PCF 1 mm vs. SLC:30 PCF 1 mm	-2.406 to -1.594	****	<0.0001
Sinus-plant:30 PCF 1 mm vs. SLC:30 PCF 3 mm	-21.17 to -20.35	****	<0.0001
Sinus-plant:30 PCF 3 mm vs. SLC:20 PCF 1 mm	14.67 to 15.49	****	<0.0001
Sinus-plant:30 PCF 3 mm vs. SLC:20 PCF 3 mm	9.064 to 9.876	****	<0.0001
Sinus-plant:30 PCF 3 mm vs. SLC:30 PCF 1 mm	13.48 to 14.30	****	<0.0001
Sinus-plant:30 PCF 3 mm vs. SLC:30 PCF 3 mm	-5.276 to -4.464	****	<0.0001
SLC:20 PCF 1 mm vs. SLC:20 PCF 3 mm	-6.016 to -5.204	****	<0.0001
SLC:20 PCF 1 mm vs. SLC:30 PCF 1 mm	-1.596 to -0.7837	****	<0.0001
SLC:20 PCF 1 mm vs. SLC:30 PCF 3 mm	-20.36 to -19.54	****	<0.0001
SLC:20 PCF 3 mm vs. SLC:30 PCF 1 mm	4.014 to 4.826	****	<0.0001
SLC:20 PCF 3 mm vs. SLC:30 PCF 3 mm	-14.75 to -13.93	****	<0.0001
SLC:30 PCF 1 mm vs. SLC:30 PCF 3 mm	-19.17 to -18.35	****	<0.0001
