# Supplementary material for: Biomechanical Analysis of Truncated Cone Implants for Maxillary Sinus Lift: An In Vitro Study on Polyurethane Laminas
Source: Bioengineering (Basel). 2025 Jan 9;12(1):53. doi: 10.3390/bioengineering12010053 (PMC11761941; doi:10.3390/bioengineering12010053)
Supplement: Supplementary file 1 [file bioengineering-12-00053-s001.zip › bioengineering-3339595-supplementary/Supplementary file S3.pdf]

Table S3. *P*-values and CI following multiple comparisons of the implant stability quotient (ISQ) values across the different experimental conditions in the buccolingual (BL) direction.

Tukey's multiple comparisons test	95.00% CI of difference	Summary	Adjusted *p*-value
Sinus-plant:20 PCF 1 mm vs. Sinus-plant:20 PCF 3 mm	-15.18 to -13.22	****	<0.0001
Sinus-plant:20 PCF 1 mm vs. Sinus-plant:30 PCF 1 mm	-4.082 to -2.118	****	<0.0001
Sinus-plant:20 PCF 1 mm vs. Sinus-plant:30 PCF 3 mm	-15.28 to -13.32	****	<0.0001
Sinus-plant:20 PCF 1 mm vs. SLC:20 PCF 1 mm	-3.582 to -1.618	****	<0.0001
Sinus-plant:20 PCF 1 mm vs. SLC:20 PCF 3 mm	-15.28 to -13.32	****	<0.0001
Sinus-plant:20 PCF 1 mm vs. SLC:30 PCF 1 mm	-7.782 to -5.818	****	<0.0001
Sinus-plant:20 PCF 1 mm vs. SLC:30 PCF 3 mm	-16.68 to -14.72	****	<0.0001
Sinus-plant:20 PCF 3 mm vs. Sinus-plant:30 PCF 1 mm	10.12 to 12.08	****	<0.0001
Sinus-plant:20 PCF 3 mm vs. Sinus-plant:30 PCF 3 mm	-1.082 to 0.8817	ns	>0.9999
Sinus-plant:20 PCF 3 mm vs. SLC:20 PCF 1 mm	10.62 to 12.58	****	<0.0001
Sinus-plant:20 PCF 3 mm vs. SLC:20 PCF 3 mm	-1.082 to 0.8817	ns	>0.9999
Sinus-plant:20 PCF 3 mm vs. SLC:30 PCF 1 mm	6.418 to 8.382	****	<0.0001
Sinus-plant:20 PCF 3 mm vs. SLC:30 PCF 3 mm	-2.482 to -0.5183	***	0.0002
Sinus-plant:30 PCF 1 mm vs. Sinus-plant:30 PCF 3 mm	-12.18 to -10.22	****	<0.0001
Sinus-plant:30 PCF 1 mm vs. SLC:20 PCF 1 mm	-0.4817 to 1.482	ns	0.7546
Sinus-plant:30 PCF 1 mm vs. SLC:20 PCF 3 mm	-12.18 to -10.22	****	<0.0001
Sinus-plant:30 PCF 1 mm vs. SLC:30 PCF 1 mm	-4.682 to -2.718	****	<0.0001
Sinus-plant:30 PCF 1 mm vs. SLC:30 PCF 3 mm	-13.58 to -11.62	****	<0.0001
Sinus-plant:30 PCF 3 mm vs. SLC:20 PCF 1 mm	10.72 to 12.68	****	<0.0001
Sinus-plant:30 PCF 3 mm vs. SLC:20 PCF 3 mm	-0.9817 to 0.9817	ns	>0.9999
Sinus-plant:30 PCF 3 mm vs. SLC:30 PCF 1 mm	6.518 to 8.482	****	<0.0001
Sinus-plant:30 PCF 3 mm vs. SLC:30 PCF 3 mm	-2.382 to -0.4183	***	0.0008
SLC:20 PCF 1 mm vs. SLC:20 PCF 3 mm	-12.68 to -10.72	****	<0.0001
SLC:20 PCF 1 mm vs. SLC:30 PCF 1 mm	-5.182 to -3.218	****	<0.0001
SLC:20 PCF 1 mm vs. SLC:30 PCF 3 mm	-14.08 to -12.12	****	<0.0001
SLC:20 PCF 3 mm vs. SLC:30 PCF 1 mm	6.518 to 8.482	****	<0.0001
SLC:20 PCF 3 mm vs. SLC:30 PCF 3 mm	-2.382 to -0.4183	***	0.0008
SLC:30 PCF 1 mm vs. SLC:30 PCF 3 mm	-9.882 to -7.918	****	<0.0001
