# Supplementary material for: Biomechanical Analysis of Truncated Cone Implants for Maxillary Sinus Lift: An In Vitro Study on Polyurethane Laminas
Source: Bioengineering (Basel). 2025 Jan 9;12(1):53. doi: 10.3390/bioengineering12010053 (PMC11761941; doi:10.3390/bioengineering12010053)
Supplement: Supplementary file 1 [file bioengineering-12-00053-s001.zip › bioengineering-3339595-supplementary/Supplementary file S4.pdf]

Table S4. *P*-values and CI following multiple comparisons of the ISQ values across the different experimental conditions in the mesiodistal (MD) direction.

Tukey's multiple comparisons test	95.00% CI of difference	Summary	Adjusted *p*-value
Sinus-plant:20 PCF 1 mm vs. Sinus-plant:20 PCF 3 mm	-15.29 to -13.31	****	<0.0001
Sinus-plant:20 PCF 1 mm vs. Sinus-plant:30 PCF 1 mm	-4.091 to -2.109	****	<0.0001
Sinus-plant:20 PCF 1 mm vs. Sinus-plant:30 PCF 3 mm	-15.19 to -13.21	****	<0.0001
Sinus-plant:20 PCF 1 mm vs. SLC:20 PCF 1 mm	-4.091 to -2.109	****	<0.0001
Sinus-plant:20 PCF 1 mm vs. SLC:20 PCF 3 mm	-15.59 to -13.61	****	<0.0001
Sinus-plant:20 PCF 1 mm vs. SLC:30 PCF 1 mm	-8.191 to -6.209	****	<0.0001
Sinus-plant:20 PCF 1 mm vs. SLC:30 PCF 3 mm	-16.99 to -15.01	****	<0.0001
Sinus-plant:20 PCF 3 mm vs. Sinus-plant:30 PCF 1 mm	10.21 to 12.19	****	<0.0001
Sinus-plant:20 PCF 3 mm vs. Sinus-plant:30 PCF 3 mm	-0.8913 to 1.091	ns	>0.9999
Sinus-plant:20 PCF 3 mm vs. SLC:20 PCF 1 mm	10.21 to 12.19	****	<0.0001
Sinus-plant:20 PCF 3 mm vs. SLC:20 PCF 3 mm	-1.291 to 0.6913	ns	0.9804
Sinus-plant:20 PCF 3 mm vs. SLC:30 PCF 1 mm	6.109 to 8.091	****	<0.0001
Sinus-plant:20 PCF 3 mm vs. SLC:30 PCF 3 mm	-2.691 to -0.7087	****	<0.0001
Sinus-plant:30 PCF 1 mm vs. Sinus-plant:30 PCF 3 mm	-12.09 to -10.11	****	<0.0001
Sinus-plant:30 PCF 1 mm vs. SLC:20 PCF 1 mm	-0.9913 to 0.9913	ns	>0.9999
Sinus-plant:30 PCF 1 mm vs. SLC:20 PCF 3 mm	-12.49 to -10.51	****	<0.0001
Sinus-plant:30 PCF 1 mm vs. SLC:30 PCF 1 mm	-5.091 to -3.109	****	<0.0001
Sinus-plant:30 PCF 1 mm vs. SLC:30 PCF 3 mm	-13.89 to -11.91	****	<0.0001
Sinus-plant:30 PCF 3 mm vs. SLC:20 PCF 1 mm	10.11 to 12.09	****	<0.0001
Sinus-plant:30 PCF 3 mm vs. SLC:20 PCF 3 mm	-1.391 to 0.5913	ns	0.9104
Sinus-plant:30 PCF 3 mm vs. SLC:30 PCF 1 mm	6.009 to 7.991	****	<0.0001
Sinus-plant:30 PCF 3 mm vs. SLC:30 PCF 3 mm	-2.791 to -0.8087	****	<0.0001
SLC:20 PCF 1 mm vs. SLC:20 PCF 3 mm	-12.49 to -10.51	****	<0.0001
SLC:20 PCF 1 mm vs. SLC:30 PCF 1 mm	-5.091 to -3.109	****	<0.0001
SLC:20 PCF 1 mm vs. SLC:30 PCF 3 mm	-13.89 to -11.91	****	<0.0001
SLC:20 PCF 3 mm vs. SLC:30 PCF 1 mm	6.409 to 8.391	****	<0.0001
SLC:20 PCF 3 mm vs. SLC:30 PCF 3 mm	-2.391 to -0.4087	***	0.0009
SLC:30 PCF 1 mm vs. SLC:30 PCF 3 mm	-9.791 to -7.809	****	<0.0001
